# Challenges of Treating Childhood Medulloblastoma in a Country With Limited Resources: 20 Years of Experience at a Single Tertiary Center in Malaysia

**DOI:** 10.1200/JGO.2015.002659

**Published:** 2016-06-15

**Authors:** Revathi Rajagopal, Sayyidatul Abd-Ghafar, Dharmendra Ganesan, Anita Zarina Bustam Mainudin, Kum Thong Wong, Norlisah Ramli, Vida Jawin, Su Han Lum, Tsiao Yi Yap, Eric Bouffet, Ibrahim Qaddoumi, Shekhar Krishnan, Hany Ariffin, Wan Ariffin Abdullah

**Affiliations:** **Revathi Rajagopal**, **Dharmendra Ganesan**, **Anita Zarina Bustam Mainudin**, **Kum Thong Wong**, **Norlisah Ramli**, **Vida Jawin**, **Su Han Lum**, **Tsiao Yi Yap**, **Shekhar Krishnan**, **Hany Ariffin**, and **Wan Ariffin Abdullah**, University of Malaya; **Sayyidatul Abd-Ghafar** and **Hany Ariffin**, University Malaya Cancer Research Institute, Kuala Lumpur, Malaysia; **Eric Bouffet**, Hospital for Sick Children, Toronto, Ontario, Canada; and **Ibrahim Qaddoumi**, St Jude Children’s Research Hospital, Memphis, TN.

## Abstract

**Purpose:**

Pediatric medulloblastoma (MB) treatment has evolved over the past few decades; however, treating children in countries with limited resources remains challenging. Until now, the literature regarding childhood MB in Malaysia has been nonexistent. Our objectives were to review the demographics and outcome of pediatric MB treated at the University Malaya Medical Center between January 1994 and December 2013 and describe the challenges encountered.

**Methods:**

Fifty-one patients with childhood MB were seen at University Malaya Medical Center. Data from 43 patients were analyzed; eight patients were excluded because their families refused treatment after surgery.

**Results:**

Headache and vomiting were the most common presenting symptoms, and the mean interval between symptom onset and diagnosis was 4 weeks. Fourteen patients presented with metastatic disease. Five-year progression-free survival (± SE) for patients ≥ 3 years old was 41.7% ± 14.2% (95% CI, 21.3% to 81.4%) in the high-risk group and 68.6% ± 18.6% (95% CI, 40.3% to 100%) in the average-risk group, and 5-year overall survival (± SE) in these two groups was 41.7% ± 14.2% (95% CI, 21.3% to 81.4%) and 58.3% ± 18.6% (95% CI, 31.3% to 100%), respectively. Children younger than 3 years old had 5-year progression-free and overall survival rates (± SE) of 47.6% ± 12.1% (95% CI, 28.9% to 78.4%) and 45.6% ± 11.7% (95% CI, 27.6% to 75.5%), respectively. Time to relapse ranged from 4 to 132 months. Most patients who experienced relapse died within 1 year. Febrile neutropenia, hearing loss, and endocrinopathy were the most common treatment-related complications.

**Conclusion:**

The survival rate of childhood MB in Malaysia is inferior to that usually reported in the literature. We postulate that the following factors contribute to this difference: lack of a multidisciplinary neuro-oncology team, limited health care facilities, inconsistent risk assessment, insufficient data in the National Cancer Registry and pathology reports, inadequate long-term follow-up, and cultural beliefs leading to treatment abandonment.

## INTRODUCTION

Malaysia is a limited-resource country that consists of two large territories, Peninsular Malaysia and East Malaysia. This multiethnic, multicultural country has a population of 30.3 million (2015 estimate),^1^ approximately 29% of which is younger age than 14 years.^1^ Malaysian public health care services are provided by the government at subsidized rates, irrespective of income or insurance status.

With improvement in socioeconomic conditions, implementation of a national immunization program, and optimal treatment of diarrheal diseases and respiratory infections, cancer has emerged as the main cause of childhood deaths in Malaysia. The National Cancer Registry was initiated in 2002, and reports are based on epidemiologic data from hospitals in Peninsular Malaysia. Statistics from East Malaysia and private practices are unavailable.^2^ A Malaysian childhood cancer survey from 2010 to 2012 found that CNS tumors are the second most common childhood malignancy (11.4%) after leukemia (46.8%). The overall incidence of CNS tumors is 9.9 per million per year (unpublished data). This rate seems to be low as a result of under-reporting of benign or non–histologically confirmed tumors, death before diagnosis, and low contribution from nonacademic institutions.

The Pediatric Hematology-Oncology Division (PHOD) at the University of Malaya Medical Center (UMMC; Kuala Lumpur, Malaysia) is a referral center for childhood cancer. The PHOD sees approximately 100 new oncology patients every year, accounting for 13% of childhood cancer diagnoses in the country annually. PHOD does not have a designated pediatric neuro-oncology team, and the number of staff members fluctuates unpredictably.

Before 1997, patients requiring radiation therapy (RT) were referred elsewhere, which delayed their treatment. In 1997, UMMC launched a radiation oncology service with two clinical radiation oncologists, and a linear accelerator was used to deliver RT. In 2002, the center implemented a radiation oncology postgraduate program and recruited seven radiation oncologists in 2013, including two with pediatric subspecialization. Conformal RT for craniospinal irradiation (CSI) was initiated in 2002, and the selection of patients was influenced by the limited number of machines available.

From 1994 to 2001, CNS tumors in Malaysia were most likely under-reported as a result of patients being transferred to other centers for surgical intervention. In 2001, UMMC established a general neurosurgical unit, followed by a pediatric neurosurgical unit with one pediatric neurosurgeon in 2008. Currently, three pediatric neurosurgeons are practicing in Malaysia. Weekly multidisciplinary team discussions involving pediatric and adult neuro-oncology staffs were implemented in 2013. The objectives of this retrospective analysis were to review the demographic data, survival outcome, prognostic factors, challenges, and limitations in the clinical management of childhood medulloblastoma (MB) at UMMC.

## METHODS

### Patients

Medical records of patients with MB treated at PHOD from January 1994 to December 2013 were retrospectively reviewed. Age, sex, ethnicity, prediagnosis symptoms, presenting signs, place and type of surgery, postoperative residual tumor, radiologic imaging, histopathology results, chemotherapy regimen, CSI dose, and outcome and treatment-related complications were recorded. The extent of surgical resection was categorized into the following four groups: gross total resection (GTR), no visible tumor remaining in the surgical field; near-total resection (NTR), removal of more than 95% but less than 100% of tumor; subtotal resection (STR), removal of more than 50% to less than 95% of tumor; and partial resection (PR), less than 50% excision of tumor. Patients with residual tumors greater than 1.5 cm^2^, metastatic disease, and/or age less than 3 years were classified as having high-risk (HR) MB. Evaluation of metastatic disease was based on the Chang classification system.^3^

### Treatment

The PHOD has used the Children’s Cancer Group (CCG) 9892 protocol since 1994 to treat children ≥ 3 years old.^4^ The protocol was designed to give reduced-dose CSI with 23.4 Gy in 13 fractions (daily fraction, 1.8 Gy) with a posterior fossa boost to patients with average-risk (AR) disease; those with HR disease received a CSI dose of 36 Gy in 20 fractions on the neuraxis. Eight weekly injections of vincristine (VCR; 1.5 mg/m^2^; maximum dose, 2 mg) were administered during CSI and followed by eight cycles of intravenous cisplatin (75 mg/m^2^) on day 1; VCR (1.5 mg/m^2^; maximum, 2 mg) on days 1, 7, and 14; and oral lomustine (75 mg/m^2^) on day 1 at 6-week intervals. Ideally, RT should be commenced within 4 weeks and no later than 7 weeks after surgery.

For patients younger than 3 years old, several protocols were used during different periods, as follows: United Kingdom Children’s Cancer Study Group (UKCCSG) 9204 (1994 to 1996),^5^ Head Start (HS) I (1997 to 2002),^6,7^ and HS II (from 2003 onward).^7,8^ These changes coincided with the availability of an autologous stem-cell transplantation (ASCT) service at UMMC.

At UMMC, ASCT is considered a treatment option for not only MB, but also metastatic Ewing sarcoma, HR neuroblastoma, relapsed extracranial germ cell tumor, and infant primitive neuroectodermal tumor. General Hospital Kuala Lumpur, UMMC, and Sime Darby Medical Center are the three pediatric oncology centers with autologous and allogeneic stem-cell or marrow transplantation programs in Malaysia.

### Statistical Methods

SPSS (version 20; SPSS, Chicago, IL) software and R statistical environment 3.2.3 (https://www.r-project.org/) were used to calculate progression-free survival (PFS) and overall survival (OS), with censoring at the time of last contact.^9^ PFS is defined as the time elapsed between treatment initiation and tumor progression. OS was calculated from the date of diagnosis to that of last follow-up or death by any cause.

## RESULTS

### Patient Demographics and Clinical Characteristics

**Fig 1:**
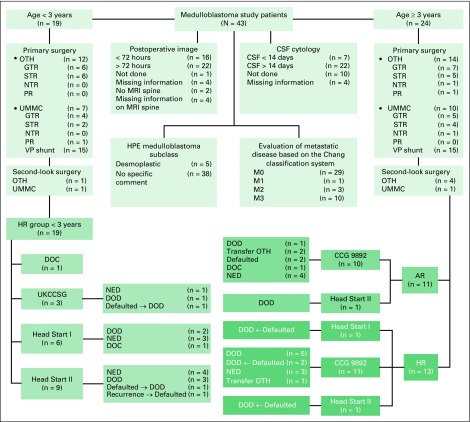
Flowchart of the pediatric medulloblastoma study. AR, average risk; CCG, Children’s Cancer Group; DOC, died of complication; DOD, dead of disease; GTR, gross total resection; HPE, histopathologic examination; HR, high risk; M0, no tumor dissemination; M1, tumor cells in the lumbar CSF; M2, cerebral meningeal thickening; M3, spinal meningeal dissemination; M4, extraneural metastases; MRI, magnetic resonance imaging; NED, no evidence of disease; NTR, near-total resection; OTH, other tertiary hospital; PR, partial resection; STR, subtotal resection; UKCCSG, United Kingdom Children’s Cancer Study Group; UMMC, University of Malaya Medical Center; VP, ventriculoperitoneal.

Fifty-one patients with MB were admitted to UMMC during the study period. Eight patients’ families declined treatment after surgery and were excluded from the study. Thus, 43 patients were studied. The median age at diagnosis was 3.5 years (range, 3 months to 15 years), and the male-to-female ratio was 1.4:1. Headache (43%) and vomiting (57%) were the most common presenting symptoms. The mean interval between onset of symptoms and diagnosis was 4 weeks (range, 1 to 20 weeks). Nineteen patients (44%) were younger than 3 years old. Ten patients had M3 disease; three had M2 disease; and one had M1 disease (Fig 1).

### Surgery and Risk Groups

All patients underwent surgical excision of the primary tumor. Twenty-two patients (51.1%) underwent GTR, two (4.7%) NTR, 17 (39.5%) subtotal resection, and two (4.7%) partial resection. Extent of resection was determined by postoperative magnetic resonance imaging (MRI) or computed tomography scans. An external ventricular drain was inserted in 11 patients, and three patients underwent ventriculostomy before definitive surgery. However, these results might be under-reported as a result of a lack of consistent data from referring institutions. Seven patients had second-look surgery, and 30 patients (69.8%) received a ventriculoperitoneal (VP) shunt (Fig 1).

Twenty-six patients (60.5%) underwent surgical resection at another institution before being referred to UMMC, including 12 patients who had residual tumor greater than 1.5 cm^2^. Seventeen patients underwent surgical resection at UMMC, seven of whom had residual tumor greater than 1.5 cm^2^. Surgery was performed by general neurosurgeons in most cases. Metastasis was infrequently investigated. CSF cytology was not investigated in 10 patients, and reports were missing for four additional patients who underwent a diagnostic lumbar puncture. Spinal MRI was not performed in two patients, and MRI reports were irretrievable for four patients. In 22 patients (51.2%), postoperative scans were performed after 72 hours (range, 4 to 30 days after surgery). Thus, risk group stratification was based on best available results for each patient at the time of diagnosis and treatment. Upon pathology review, one diagnosis was revised to MB from ependymoma after tumor recurrence. This patient was treatment naïve before relapse, so the patient was subsequently managed as having a newly diagnosed HR MB. Histology subclass was reported as desmoplastic in five patients; the remaining 38 patients had no record of histology subclass (Fig 1).

### Treatment of Patients Age 3 Years or Older: RT and Chemotherapy

Of the children age ≥ 3 years, three patients were enrolled onto the HS protocol because their families refused RT. The patients were 4, 5, and 6 years old. Two of these patients discontinued treatment after one course of chemotherapy and subsequently died of progressive disease. The other patient received five courses of chemotherapy; local and leptomeningeal recurrence was then detected before ASCT, and the patient received palliative RT (Table 1). The remaining patients received CSI at a median time of 5 weeks after surgery (range, 2 to 9 weeks). CSI was delayed in eight patients, one of whom received a first course of CCG 9892 chemotherapy (Table 1).

**Table 1:**
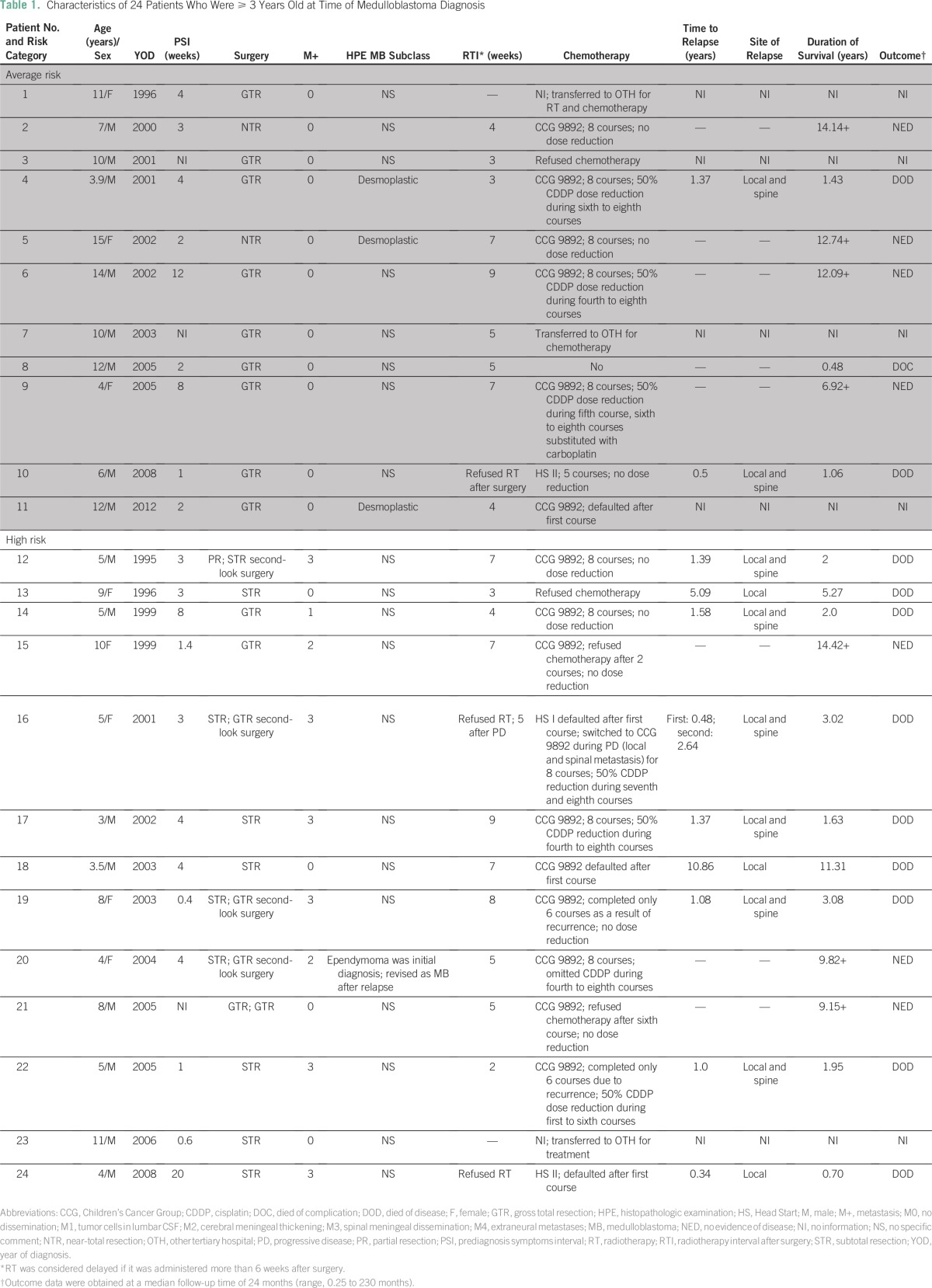
Characteristics of 24 Patients Who Were ≥ 3 Years Old at Time of Medulloblastoma Diagnosis

After CSI, 15 patients received CCG 9892 chemotherapy (Table 1). The families of two patients refused chemotherapy after the first course, one of whom experienced local recurrence 10 years later and underwent complete excision that was complicated by severe neurologic impairment requiring tracheostomy; no salvage chemotherapy was given. One patient remains in complete remission despite refusing chemotherapy after two courses. Among three patients who received six cycles of chemotherapy, two suffered local and leptomeningeal recurrence after the sixth course and received palliative oral etoposide. Nine patients completed eight cycles of chemotherapy, three of whom experienced local and leptomeningeal recurrence (Table 1).

### Treatment of Patients Younger Than 3 Years Old: Chemotherapy Only

Of the 19 patients younger than age 3 years, one died of a postoperative complication, and 18 received chemotherapy (Fig 1 and Table 2). One family refused CSI after completion of UKCCSG chemotherapy, and that patient is still in complete remission. The other two patients treated on the UKCCSG protocol experienced tumor recurrence after the second and third cycles of chemotherapy, respectively. One patient received CSI (35.2 Gy with boost) followed by two courses of CCG 9892 chemotherapy, but treatment failed. Two patients on the HS I protocol did not undergo ASCT; one of these patients died of *Candida* sepsis after the second course of chemotherapy, and the other died of disease recurrence after the fifth course without salvage treatment. Nine patients were treated per the HS II protocol, but four of these patients did not receive ASCT. Three patients experienced tumor recurrence after the second, third, and fourth courses, respectively, and none received salvage treatment. One family refused ASCT and opted for traditional medicine. This child experienced MB recurrence after 2 months and was referred to palliative care. Only nine infants received myeloablative chemotherapy with ASCT (Table 2).

**Table 2:**
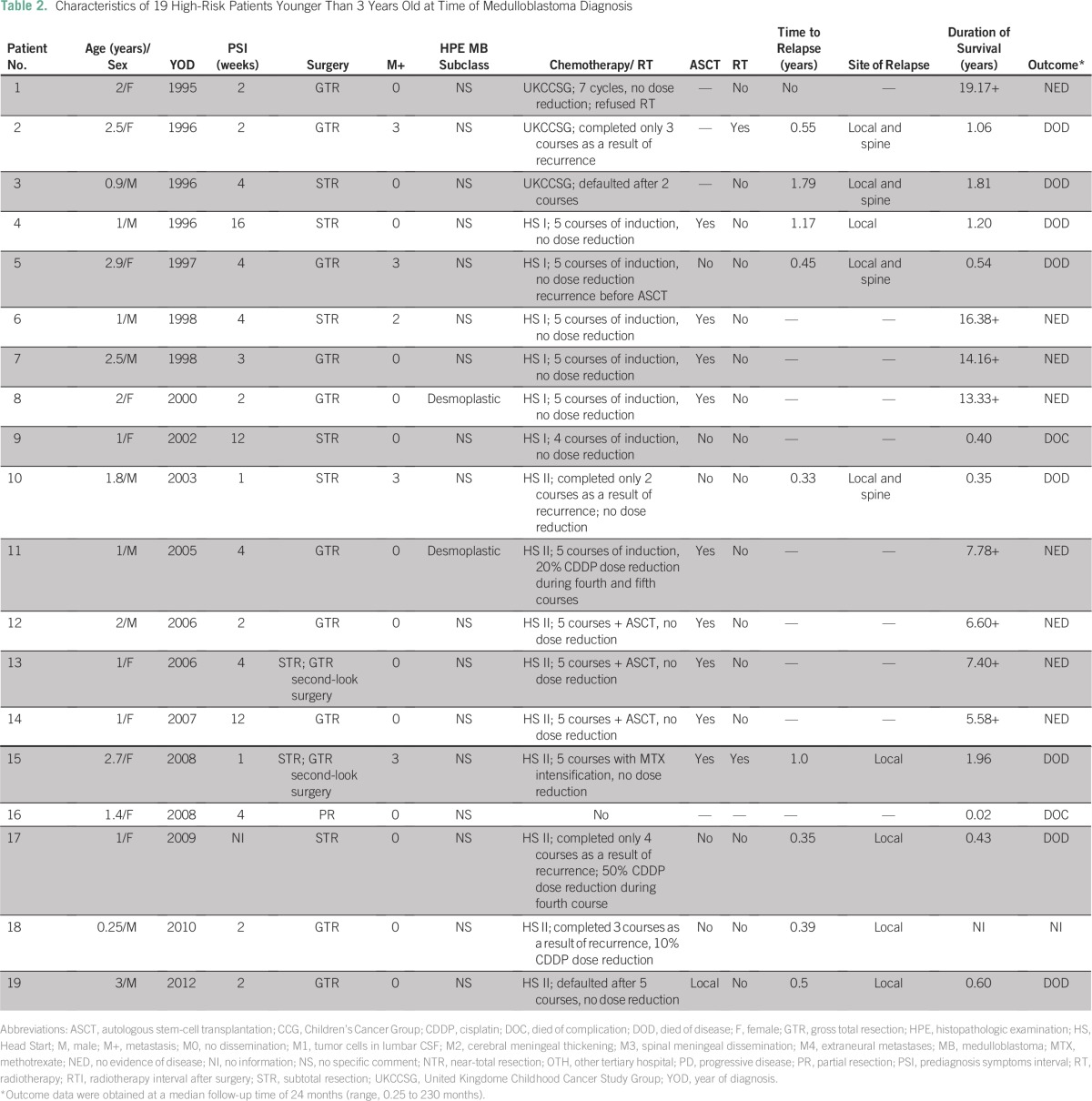
Characteristics of 19 High-Risk Patients Younger Than 3 Years Old at Time of Medulloblastoma Diagnosis

### Outcome

The median follow-up time of survivors was 12 years (range, 5.75 to 19.17 years). MB recurred in 19 patients, including six who discontinued treatment. The sites of relapse were local (n = 7) and local with spinal dissemination (n = 12). Median time to relapse was 12 months (range, 4 to 132 months), and most patients died within 1 year of relapse.

**Fig 2:**
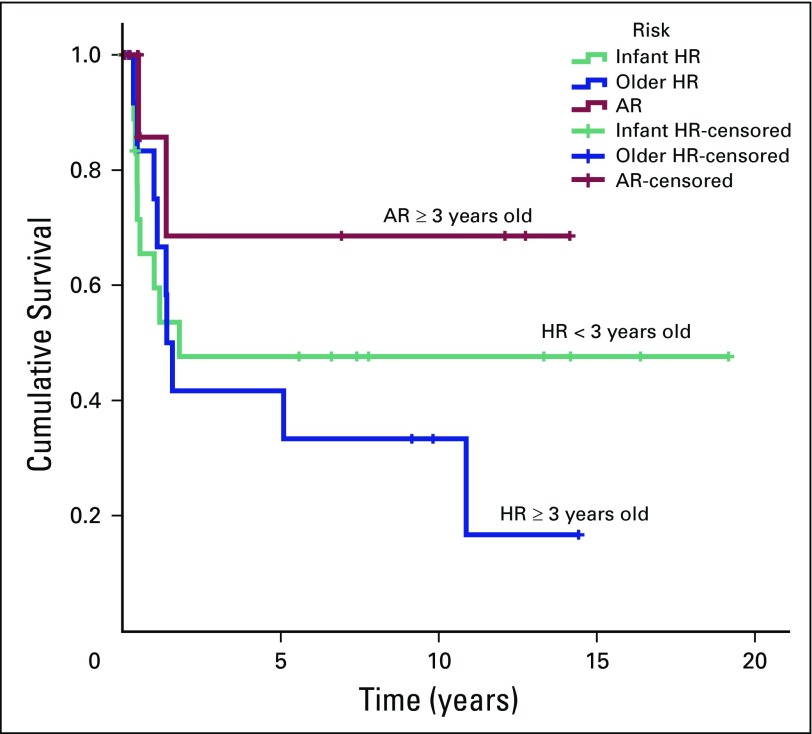
Progression-free survival of pediatric patients with average-risk (AR) and high-risk (HR) medulloblastoma treated from 1994 to 2013.

**Fig 3:**
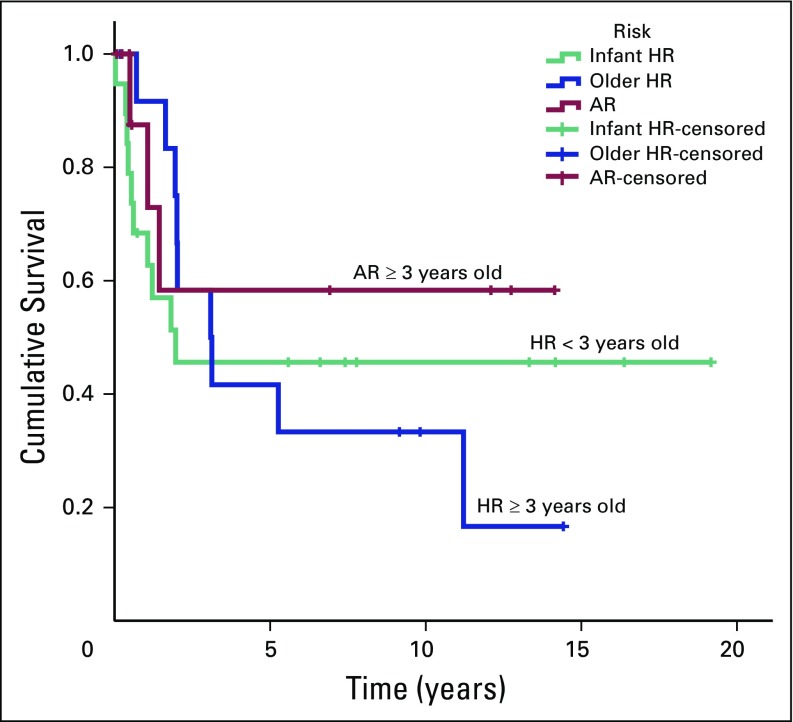
Overall survival of pediatric patients with average-risk (AR) and high-risk (HR) medulloblastoma treated from 1994 to 2013.

The 5-year PFS rates (± SE) for older children in the HR group, patients in the AR group, and younger children in the HR group were 41.7% ± 14.2% (95% CI, 21.3% to 81.4%), 68.6% ± 18.6% (95% CI, 40.3% to 100%), and 47.6% ± 12.1% (95% CI, 28.9% to 78.4%), respectively. The 5-year OS rates (± SE) were 41.7% ± 14.2% (95% CI, 21.3% to 81.4%) in older patients in the HR group, 58.3% ± 18.6% (95% CI, 31.3% to 100%) in patients in the AR group, and 45.6% ± 11.7% (95% CI, 27.6% to 75.5%) for children younger than age 3 years (Figs 2 and 3). In this study, age less than 3 years did not influence the outcome. In addition, it is difficult to conclude the role of residual tumor greater than 1.5 cm^2^ and distant metastasis in relation to prognosis as a result of inconsistent risk assessments among patients. Fifteen survivors are still being followed, including seven patients from the older age group and eight patients from the younger age group, including one patient with M2 disease. None of the eight survivors in the younger group required RT.

### Treatment-Related Complications

In the group of older children, the most common treatment-related complication was high-frequency sensorineural hearing loss. Hearing tests were performed routinely in all patients before commencement of treatment. Eleven patients had evidence of moderate to severe hearing loss during treatment surveillance, and seven patients required cisplatin dose adjustment. No records were available on hearing aid usage. One patient had pre-existing hearing loss before the first course of chemotherapy. In patients who received chemotherapy on the CCG 9892 protocol, hearing loss occurred as early as after the second course of chemotherapy (median time to hearing loss, fourth course). We did not find any correlation between the location of the tumor (midline or lateral) and the occurrence of hearing loss. Two patients suffered reversible VCR-induced neuropathy with paralytic ileus and ptosis. VCR was then omitted from the regimen until full recovery of the clinical signs. The incidence of neutropenic fever was low in the older children. Three episodes of sterile febrile neutropenia (FN), two episodes of *Pseudomonas* pneumonia, and one episode each of *Escherichia coli* urinary tract infection and methicillin-resistant *Staphylococcus epidermidis* meningitis were observed. One patient died during CSI secondary to *Acinetobacter baumannii* infection of a VP shunt.

Thirty-six episodes of FN were seen in 15 younger children on the HS protocols; three of these patients were admitted for a single FN episode, and 12 experienced two to four episodes. Most FN episodes (52.8%) had a positive blood culture, and the documented organisms included methicillin-resistant *S epidermidis*, *Candida albicans*, *E coli*, methicillin-sensitive *Staphylococcus aureus*, *A baumannii*, extended-spectrum β-lactamases, *Klebsiella pneumoniae*, *Staphylococcus saprophyticus*, *Streptococcus mitis*, and *Pseudomonas aeruginosa*. One patient recovered from *S mitis* infective endocarditis, and two patients required admission to the pediatric intensive care unit for hemodynamic instability as a result of methicillin-sensitive *S aureus* and *P aeruginosa* infection, respectively. One death occurred secondary to disseminated *Candida* infection. All patients required blood transfusions, especially platelet support during chemotherapy. Severe myelotoxicity (eg, neutropenia, thrombocytopenia) was observed during the HS I and II protocols, despite prophylactic administration of granulocyte colony-stimulating factor. Five infants with midline tumors suffered moderate to severe high-frequency sensorineural hearing loss, and two required cisplatin dose adjustment.

Endocrine insufficiency was the most common long-term sequela in older patients. Six (85%) of seven survivors and one patient with relapsed MB had endocrine insufficiencies. Full-panel endocrine tests were performed every 6 months after completion of treatment, and hypothyroidism was the first finding in these patients. All of these patients required thyroxine replacement after 18 months of CSI. Neuropsychology and cognitive testing were not performed because no psychologist was available. Neurocognitive assessments were based on academic performance during outpatient follow-up. Poor academic performance and short attention span were noted in four older patients and two younger patients.

## DISCUSSION

Survival of childhood MB has dramatically improved over the past few decades as a result of the adoption of a multimodal treatment approach.^10^ Recent clinical trials in high-income countries have shown that patients with AR MB who receive reduced-dose CSI and adjuvant chemotherapy experience 5-year event-free survival (EFS) greater than 80%, and those with HR MB who receive standard-dose CSI and adjuvant chemotherapy experience 5-year EFS of 66% to 70%.^3,11,12^ The outcome of MB in low-income countries varies based on the availability of health care resources and choice of chemotherapy regimens. At UMMC, the 5-year OS rates for older patients with HR and AR disease were 41.7% and 58.3%, respectively; in younger patients, this rate was 45.6%. These rates are low compared with those in high-income countries^7,11-16^ (Table 3), and several reasons may explain this difference.

**Table 3:**
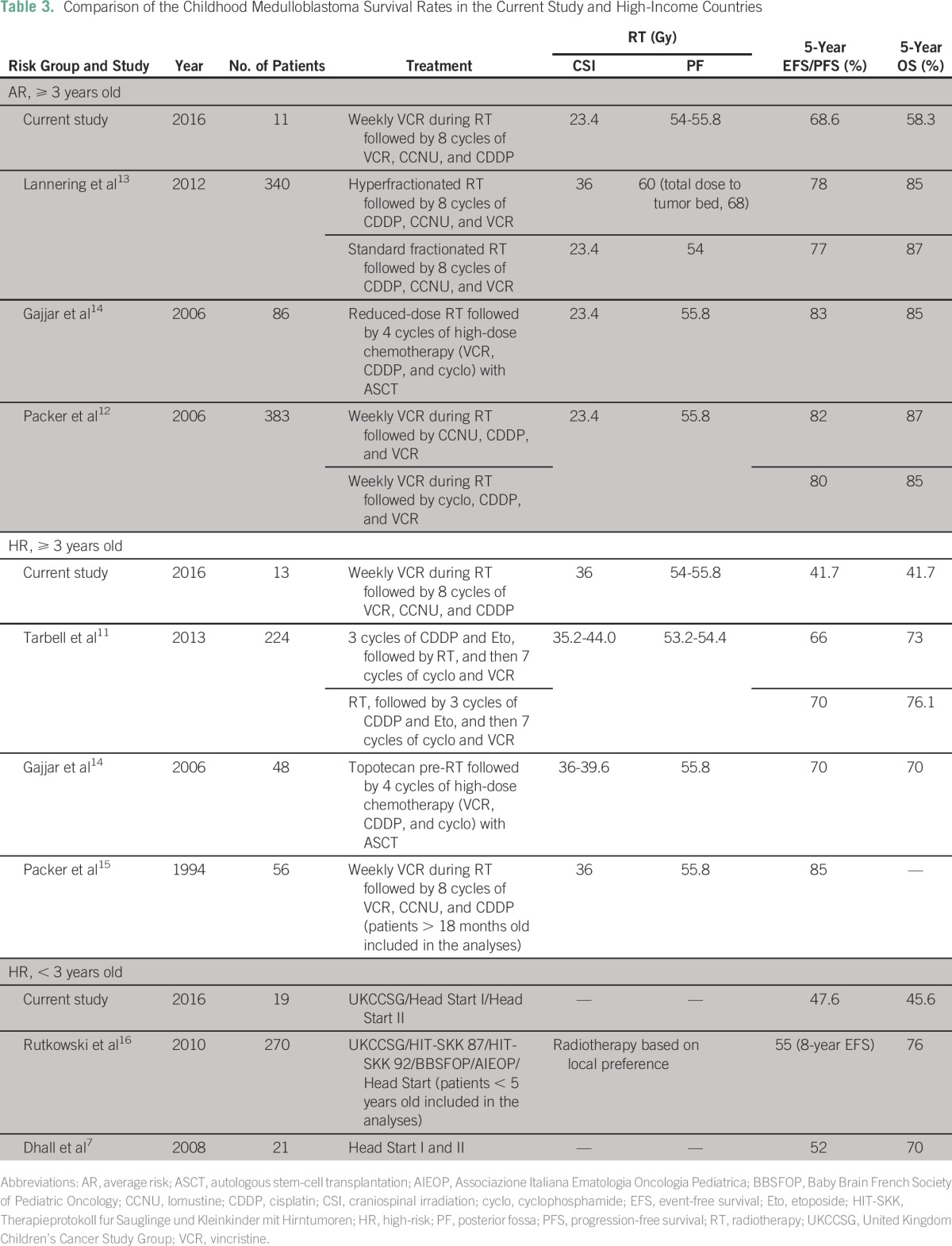
Comparison of the Childhood Medulloblastoma Survival Rates in the Current Study and High-Income Countries

Until the early 2000s, most patients with MB treated at UMMC underwent their initial surgical intervention elsewhere because of the lack of pediatric neurosurgical support at UMMC, and most of those interventions were performed by general neurosurgeons. As a result, only 55.8% of pediatric patients had GTR or NTR, and 70% required VP shunt insertion. Our GTR + NTR rate was inferior to that in other developing countries. Menon et al^17^ reported a GTR + NTR rate of 75% in India, and only 29% of their patients required a VP shunt.

The limited number of linear accelerators, frequent machine breakdowns, long waiting lists, and a lack of trained pediatric radiotherapists were significant barriers to commence RT in a timely manner (ie, RT should commence within 4 weeks of surgical resection of MB). Therefore, clinical management was fragmented, and adherence to protocol guidelines was lax. In our series, only seven patients began RT by 4 weeks; four patients commenced RT at 4 to 6 weeks; and eight patients started RT after 6 weeks after surgery. One patient received postoperative chemotherapy while waiting for RT to prevent MB recurrence and dissemination. Although the UMMC health care facilities have improved over the years, further development is required, especially development of a well-structured multidisciplinary team approach.

The timing of imaging examinations after surgery is important for assessing residual disease and leptomeningeal dissemination. Postoperative imaging of children is often delayed because general anesthesia may be required to obtain MRIs, and longer imaging time is needed for brain and spine pre- and postcontrast sequences. In addition, inconsistent assessments (eg, absence of spinal MRIs and/or CSF analysis) may have resulted in incorrect risk stratification.

Besides risk assessment, the lack of neuropathology expertise was a limitation as evidenced by the absence of tumor histology subclass in most reports. Diffuse anaplasia indicates poor prognosis, and patients with that histology subtype may benefit from more intensive treatment. Similarly, infants with desmoplastic MB and MB with extensive nodularity experience excellent outcome with chemotherapy alone.^18,19^ Recently, several molecular and genetic tests have been developed that may improve risk stratification.^18,19^ The UMMC Biobank started tumor tissue banking in 2012, which will be useful for future molecular and genetic studies.

Chemotherapy approaches for children younger than 3 years old varied, and the numbers were too small to compare across protocols. The 5-year OS and EFS rates were acceptable when compared with those of other developing countries, but this response might not reflect long-term disease control. This group received definitive surgery and chemotherapy without delay. More intensive chemotherapy with ASCT and better supportive care during the mid- to late 2000s may have contributed to these promising results. Treatment of the older children on CCG 9892 varied by dose and the number of courses. Therefore, some patients may have received suboptimal treatment.

A literature review showed that MB survivors have multiple long-term morbidities (eg, physical disability, endocrinopathy, hearing loss, visual impairment).^20,21^ The majority of our survivors (85%) have experienced endocrine insufficiency and required hormonal replacement. Apart from treating endocrinopathy, no formal long-term follow-up was scheduled at UMMC.

Cultural barriers and a lack of parental awareness influence treatment adherence in Malaysia. Reasons for treatment abandonment were intolerance to chemotherapy adverse effects, sepsis, poor social support during prolonged hospitalization, lack of housing facilities for families from distant areas, and cultural beliefs that traditional medicine is superior. In our series, eight patients declined treatment after surgery, three were lost to follow-up, and seven refused further treatment in the midst of chemotherapy. One Malaysian study found that 25% of parents sought help from traditional healers while their children were receiving cancer treatment in the hospital. The patients were given cactus juice, blessed water from holy places, horse milk, and ginseng.^22^ Financial issues were not a major factor in treatment refusal because the health care services are subsidized by the government. Since 2013, most families have received free accommodations at UMMC to reduce the burden of traveling and facilitate treatment without interruption.

Pediatric neuro-oncology is still an evolving subspecialty in Malaysia. Several steps to overcome the gaps and challenges must be addressed to improve the outcome of children with MB. Coordinated multidisciplinary pediatric neuro-oncology teams with better health care facilities, standardized treatment protocols, accurate disease staging and risk stratification, detailed pathology reports, formal assessment of long-term morbidity, and implementation of a national cancer registry require further attention from pediatric hematology-oncology units. Furthermore, a twinning program and research collaborations with developed countries should be considered to improve the outcome of childhood MB in the future. Such initiatives have shown excellent outcomes in childhood leukemia at UMMC through the Malaysia-Singapore 2003 research collaboration,^23^ and this approach has significantly improved the survival of patients with MB in Jordan.^10^
